# Towards Multifractality through an Ernst-Type Potential in Complex Systems Dynamics

**DOI:** 10.3390/e25081149

**Published:** 2023-07-31

**Authors:** Vlad Ghizdovat, Oana Rusu, Mihail Frasila, Cristina Marcela Rusu, Maricel Agop, Decebal Vasincu

**Affiliations:** 1 Department of Biophysics and Medical Physics, “Grigore T. Popa” University of Medicine and Pharmacy, 700115 Iasi, Romania; vlad.ghizdovat@umfiasi.ro; 2 Faculty of Material Science and Engineering, “Gheorghe Asachi” University of Iasi, 700050 Iasi, Romania; oana.rusu@academic.tuiasi.ro; 3 Faculty of Physics, “Alexandru Ioan Cuza” University of Iasi, 700050 Iasi, Romania; frasilamihail@yahoo.com; 4 Department of Physics, “Gheorghe Asachi” Technical University of Iasi, 700050 Iasi, Romania; cristina.rusu@tuiasi.ro; 5 Romanian Scientists Academy, 54 Splaiul Independentei, 050094 Bucharest, Romania; 6 Faculty of Dental Medicine, University of Medicine and Farmacy Grigore T. Popa Iași, 700050 Iasi, Romania; decebal.vasincu@umfiasi.ro

**Keywords:** scale relativity theory, space–time theory, SL (2R) group, Ernst potential, multifractality, complex systems

## Abstract

Some possible correspondences between the Scale Relativity Theory and the Space–Time Theory can be established. Since both the multifractal Schrödinger equation from the Scale Relativity Theory and the General Relativity equations for a gravitational field with axial symmetry accept the same SL(2R)-type invariance, an Ernst-type potential (from General Relativity) and also a multi-fractal tensor (from Scale Relativity) are highlighted in the description of complex systems dynamics. In this way, a non-differentiable description of complex systems dynamics can become functional, even in the case of standard theories (General Relativity and Quantum Mechanics).

## 1. Introduction

Although differentiability is most commonly used in the description of complex systems dynamics [1,2,3,4], some natural nonlinear phenomena (such as states transitions, self-structuring, chaos, etc.) would greatly benefit from non-differentiability as a natural way of describing such dynamics. For example, in interactions found in various fluids, gas, and plasma, the trajectory of any particle, between two successive collisions, is a straight line (a continuous and differentiable curve). At the impact (collision) point, this curve becomes non-differentiable. As a consequence, the set of all impact points is equivalent to a fractal set [3]. 

Non-differentiability employed in the description of complex systems dynamics can be accomplished either through fractional derivatives formalism or through the Scale Relativity Theory (SRT) [5,6,7,8,9]. Regarding this theory, the description of complex systems dynamics can be achieved by employing either mono-fractal manifolds (dynamics in a single fractal dimension [7]) or multifractal manifolds (dynamics in multiple simultaneous fractal dimensions [7,9])

It follows that, in the context of SRT, any physical quantity (used in the description of complex systems dynamics) will depend on space–time coordinates, as well as on a scale resolution. This leads us to a description of these dynamics by means of strictly non-differentiable mathematical functions. We must mention that any non-differentiable function will operate as the limit of a family of mathematical functions. This function is non-differentiable at null-scale resolution and differentiable at non-null-scale resolution [5,6,7,8,9]. These non-differentiable functions display a self-similarity property. In our opinion, such a property could be associated to holographic-type behaviors in the description of complex systems dynamics [6,8,9].

The concept of holography can be found in modern space–time theories, in the form of the holographic principle [10]. By employing this principle, scientists are trying to find correlations between the Space–Time Theory (i.e., General Relativity) and the Quantum Theory (in order to determine a unified theory of physical interactions). 

In the present paper, correlations between SRT and General Relativity (for the case of the axially symmetric field) are given. 

## 2. A Short Reminder on Schrödinger- and Madelung-Type Scenarios in the Description of Complex Systems Dynamics

It is a known fact that the dynamics of complex systems in the SRT [6,7,8,9] can be described through the multifractal Schrödinger equation—the Schrödinger-type scenario:

(1) $$\lambda^{2}{\left(dt\right)}^{\left[\frac{4}{f\left(\alpha \right)}\right]-2}\partial_{l}\partial^{l}\Psi +i\lambda {\left(dt\right)}^{\left[\frac{2}{f\left(\alpha \right)}\right]-1}\partial_{t}\Psi =0$$

where

(2) $$\partial_{t}=\frac{\partial }{\partial t},\;{\;\partial }_{l}=\frac{\partial }{\partial x^{l}}\;,\;{\;\partial }_{l}\partial^{l}=\frac{\partial^{2}}{\partial x_{l}^{2}}$$

In Equations (1) and (2), Ψ is a state function; $x^{l}$ is the multifractal spatial coordinate; t is the non-multifractal temporal coordinate with the role of an affine parameter of the multifractal curves; λ  is a diffusion-type constant associated to the differentiable–non-differentiable transitions in the dynamics of complex systems; dt is the scale resolution; and $f\left(\alpha \right)$ is the singularity spectrum of order α, $\alpha =\alpha \left(D_{F}\right)$, where $D_{F}$ is the fractal dimension of the motion curves. In such a context, it is possible, through $f\left(\alpha \right)$, to identify the areas of the complex system’s dynamics that are characterized by a certain fractal dimension (mono-fractal complex systems dynamics), and also to identify the number of areas for which their fractal dimensions can be found in a value interval (multifractal complex systems dynamics). Regarding the fractal dimensions, several definitions can be used, such as the Kolmogorov fractal dimension or Hausdorff–Besicovitch fractal dimension [5,6,7,8,9].

By choosing Ψ of the form

(3) $$\Psi =\sqrt{\rho }e^{is}$$

where $\sqrt{\rho }$ is the amplitude, and s is the phase, and introducing the real velocity fields ($V_{D}^{i}$—differentiable velocity field, $V_{F}^{i}$—non-differentiable velocity field):

(4) $$V_{D}^{i}=2\lambda (dt{)}^{\left[\frac{2}{f\left(\alpha \right)}\right]-1}\partial^{i}s$$

(5) $$V_{F}^{i}=i\lambda (dt{)}^{\left[\frac{2}{f\left(\alpha \right)}\right]-1}\partial^{i}\ln\rho$$

Equation (1) is reduced to the multifractal hydrodynamic equation system, i.e., the Madelung-type scenario:

(6) $$\partial_{t}V_{D}^{i}+V_{D}^{l}\partial_{l}V_{D}^{i}=-\partial^{i}Q$$

(7) $$\partial_{t}\rho +\partial_{l}\left(\rho V_{D}^{l}\right)=0$$

with Q the multifractal specific potential:

(8) $$Q=-2\lambda^{2}(dt{)}^{\left[\frac{4}{f\left(\alpha \right)}\right]-2}\frac{\partial^{l}\partial_{l}\sqrt{\rho }}{\sqrt{\rho }}=-V_{F}^{i}V_{F}^{i}-\frac{1}{2}\lambda (dt{)}^{\left[\frac{2}{f\left(\alpha \right)}\right]-1}\partial_{l}V_{F}^{l}$$

Equation (6) corresponds to the multifractal specific momentum conservation law. Equation (7) corresponds to the multifractal state density conservation law. Moreover, through (8), the multifractal specific force results in:

(9) $$F^{i}=-\partial^{i}Q=-2\lambda^{2}(dt{)}^{\left[\frac{4}{f\left(\alpha \right)}\right]-2}\partial^{i}\frac{\partial^{l}\partial_{l}\sqrt{\rho }}{\sqrt{\rho }}$$

which is a measure of the multifractality of the motion curves of the dynamics.

The main consequences of the multifractal hydrodynamic equation systems are given in [11,12].

Now, by employing the multifractal tensor:

(10) $${\hat{\tau }}^{il}=2\lambda^{2}{\left(dt\right)}^{\left[\frac{4}{f\left(\alpha \right)}\right]-2}\rho \partial^{i}\partial^{l}\ln\rho$$

Equation (9) takes the form of a multifractal equilibrium equation

(11) $$\rho \partial^{i}Q=\partial_{l}{\hat{\tau }}^{il}$$

Since the multifractal tensor ${\hat{\tau }}^{il}$ can also be written in the form:

(12) $${\hat{\tau }}^{il}=\eta \left(\partial_{l}V_{F}^{i}+\partial_{i}V_{F}^{l}\right)$$

with

(13) $$\eta =\lambda {\left(dt\right)}^{\left[\frac{2}{f\left(\alpha \right)}\right]-1}\rho$$

a multifractal constitutive law for a multifractal “viscous fluid” can be applied. In this way, it is possible to provide an original interpretation of coefficient η  as a multifractal dynamic viscosity of the multifractal fluid.

## 3. Riemannian-Type Geometries Associated to the Multifractal Tensor ${\hat{\tau }}^{il}$

Due to the fact that the multifractal tensor ${\hat{\tau }}^{il}$ becomes fundamental in the definition of a constitutive material law, let us specify some of its properties.

The eigenvalues (by means of its characteristic/secular equation) of the ${\hat{\tau }}^{il}$ multifractal tensor imply the cubic equation [13,14]:

(14) $$a_{0}x^{3}+3a_{1}x^{2}+3a_{2}x+a_{3}=0$$

If we admit that (14) has real roots [15,16], then these roots can be written as:

(15) $$x_{1}=\frac{h+h^{*}k}{1+k}\;x_{2}=\frac{h+\varepsilon h^{*}k}{1+\varepsilon k}\;x_{3}=\frac{h+\varepsilon^{2}h^{*}k}{1+\varepsilon^{2}k}\;$$

In (15), h, $h^{*}$ are the roots of Hessian

$$H={\left(a_{0}a_{3}-a_{1}a_{2}\right)}^{2}-4\left(a_{0}a_{2}-a_{1}^{2}\right)\left(a_{1}a_{3}-a_{2}^{2}\right)$$

and $\varepsilon \equiv \left(-1+i\sqrt{3}\right)/2$ is the cubic root of unity $\left(i=\sqrt{-1}\right)$. We note that all the values of variables h, $h^{*},$ and k can be organized in a simple transitive group with real parameters. This simple transitive group can be highlighted through Riemann-type spaces associated with the cubic (14). The basis of this approach is the fact that the simply transitive group with real parameters [9,15,16]:

(16) $$x_{l}↔\frac{ax_{l}+b}{cx+d}\;,\;l=1,2,3\;a,b,c,d\in R$$

where $x_{l}$ are the roots of the cubic (14), induces the simply transitive group in the quantities h, $h^{*},$ and k (as a unimodular factor), whose actions are:

(17) $$h↔\frac{ah+b}{ch+d}\;h^{*}↔\frac{ah^{*}+b}{ch^{*}+d}\;k↔\frac{ch^{*}+d}{ch+d}k$$

The structure of this group is of a SL(2,R) type, and we take it in the standard form. Due to its simple transitivity property, the generators can be easily found as the components of the Cartan landmark of the group as before [17,18], from relation

(18) $$\begin{array}{ll} d(f) & \equiv \sum \;\frac{\partial f}{\partial x^{k}}dx^{k}\\ & =\left[\omega^{1}\left(h^{2}\frac{\partial }{\partial h}+h^{*2}\frac{\partial }{\partial h^{*}}k\frac{\partial }{\partial k}\right)\right.\\ & \left.+2\omega^{2}\left(h\frac{\partial }{\partial h}+h^{*}\frac{\partial }{\partial h^{*}}\right)+\omega^{3}\left(\frac{\partial }{\partial h}+\frac{\partial }{\partial h^{*}}\right)\right](f) \end{array}$$

where $\omega^{k}$ are the three differential forms that give the components of the Cartan coframe that can be found from the algebraic system

(19) $$\begin{array}{ll} & dh=\omega^{1}h^{2}+2\omega^{2}h+\omega^{3}\\ & d\bar{h}=\omega^{1}h^{*2}+2\omega^{2}h^{*}+\omega^{3}\\ & dk=\omega^{1}k\left(h-h^{*}\right) \end{array}$$

Therefore, we immediately have both the infinitesimal generators and the Cartan coframe, by identification of the right side of Equation (18) with the standard scalar product of the structure SL(2,R) [15,16]

(20) $$\omega^{1}B_{3}+\omega^{3}B_{1}-2\omega^{2}B_{2}$$

so that

(21) $$\begin{array}{ll} B_{1} & =\frac{\partial }{\partial h}+\frac{\partial }{\partial h^{*}},\;B_{2}=h\frac{\partial }{\partial h}+h^{*}\frac{\partial }{\partial h^{*}}\\ B_{3} & =h^{2}\frac{\partial }{\partial h}+h^{*2}\frac{\partial }{\partial h^{*}}+\left(h-h^{*}\right)k\frac{\partial }{\partial k} \end{array}$$

and

(22) $$\begin{array}{ll} \omega^{1} & =\frac{dk}{\left(h-h^{*}\right)k},2\omega^{2}=\frac{dh-dh^{*}}{h-h^{*}}-\frac{h+h^{*}}{h-h^{*}}\frac{dk}{k}\\ \omega^{3} & =\frac{hdh^{*}-h^{*}dh}{h-h^{*}}+\frac{hh^{*}}{h-h^{*}}\frac{dk}{k} \end{array}$$

In real variables, given by $h\equiv u+iv,k=e^{i\Phi }$, these equations can be written as

(23) $$\begin{array}{ll} B_{1} & =\frac{\partial }{\partial u},B_{2}=u\frac{\partial }{\partial u}+v\frac{\partial }{\partial v}\\ B_{3} & =\left(u^{2}-v^{2}\right)\frac{\partial }{\partial u}+2uv\frac{\partial }{\partial v}+2v\frac{\partial }{\partial \phi }\\ \omega^{1} & =\frac{d\phi }{2v},\omega^{2}=\frac{dv}{v}-\frac{u}{v}d\phi \\ \omega^{3} & =\frac{u^{2}+v^{2}}{2v}d\phi +\frac{vdu-udv}{v} \end{array}$$

In order to respect the historical truth, we must mention that in the original article from 1938, Barbilian does not work with these differential forms, but with the absolutely invariant differentials [19]

(24) $$\omega^{1}=\frac{dh}{\left(h-h^{*}\right)k},\omega^{2}=-i\left(\frac{dk}{k}-\frac{dh+dh^{*}}{h-h^{*}}\right),\omega^{3}=-\frac{kdh^{*}}{h-h^{*}}$$

or, in real variables, with the differential forms

(25) $$\begin{array}{ll} & \Omega^{1}=d\phi +\frac{du}{v}\\ & \Omega^{2}=\cos\phi \frac{du}{v}+\sin\phi \frac{dv}{v}\\ & \Omega^{3}=-\sin\phi \frac{du}{v}+\cos\phi \frac{dv}{v} \end{array}$$

which highlights a three-dimensional Lorentz structure of the metric space, to which they refer. The differential forms (25) actually represent a Bücklund transformation of the hyperbolic plane connection [20]. Moreover, it can be seen easily that the infinitesimal generators of the group differ from the Beltrami operators characteristic of the Lobachevski geometry in the Poincaré representation only by the component 2v∂/∂ϕ in the expression of the third operator. If we calculate the invariant metric of the group, either from components (22) of the coframe or from (25) as the Lorentz metric, we will obtain the known expression

(26) $$ds^{2}={\left(d\phi +\frac{du}{v}\right)}^{2}+\frac{(du{)}^{2}+(dv{)}^{2}}{v^{2}}$$

in which we recognize the Beltrami metric, to which is added (with a negative sign) the square of the differential form whose cancellation defines the parallelism angle in the Lobachevski plane, i.e., the connection form [21]. Let us note that, according to the above, because through relation

$$\omega^{2}=0⟺d\phi =-\frac{du}{v}$$

a direction parallelism of the Lobachevski plane in a Levi-Civita sense can be defined, the metric (26) can be reduced to its Poincaré representation, i.e.,

(27) $$ds^{2}=\frac{dhdh^{*}}{{\left(h-h^{*}\right)}^{2}}=\frac{(du{)}^{2}+(dv{)}^{2}}{v^{2}}$$

Let us note that metric (27) can be considered as a Lagrangian (geodesic Lagrangian), and it can provide a momentum vector. Now, the general methodology from [16,17,20] states that the projection of this vector on a Killing vector of the metric (27) is a quantity that conserves along the geodesics. In this way, these quantities gain direct physical meaning.

We must mention that this formalism can be applied to any scale resolution of a complex system’s dynamics, i.e., we are discussing here differential geometries of a multifractal-type, which evidently implies multifractal metrics.

## 4. Harmonic Mappings from the Usual Space to the Hyperbolic One

Let us assume that the complex system’s dynamics are described by the field variables $\left(Y^{j}\right)$, for which, at any scale resolution, the following metric was discovered:

(28) $$h_{ij}dY^{i}dY^{j}$$

in the usual space of the metric:

(29) $$\gamma_{\alpha \beta }dX^{\alpha }dX^{\beta }$$

Then, the field equations result by using a variational principle applied to the multifractal Lagrangian:

(30) $$L=\gamma^{\alpha \beta }h_{ij}\frac{dY^{i}dY^{j}}{\partial X^{\alpha }\partial X^{\beta }}$$

In our case, (28) is given by (27). Now, for the variational principle

(31) $$\delta \int L\sqrt{\gamma }d^{3}x=0$$

applied to the Lagrangian (30), the multifractal Euler equations are

(32) $$\left(h-h^{*}\right)\nabla \left(\nabla h\right)=2{\left(\nabla h\right)}^{2}\;\left(h-h^{*}\right)\nabla \left(\nabla h^{*}\right)=2{\left(\nabla h^{*}\right)}^{2}$$

and admit the solution

(33) h=cosh⁡Φ2−sinh⁡Φ2e−iαcosh⁡Φ2+sinh⁡Φ2e−iα, α∈R

In (33), α is real and arbitrary, as long as Φ2 is the solution of a multifractal Laplace equation for the free space, such that

(34) ∇2Φ2=0

**Figure 1:**
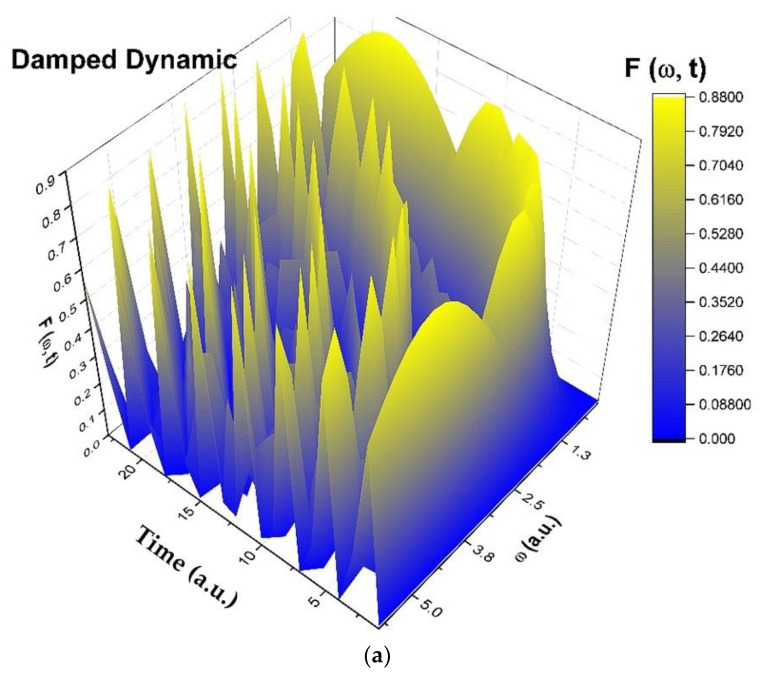

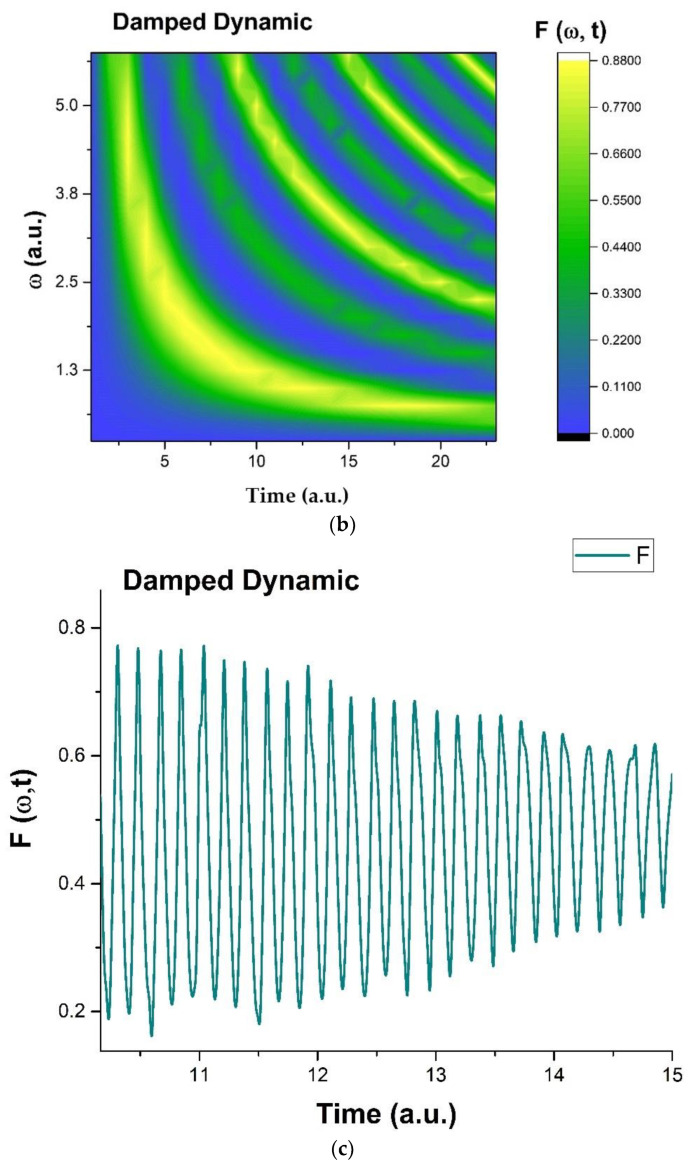

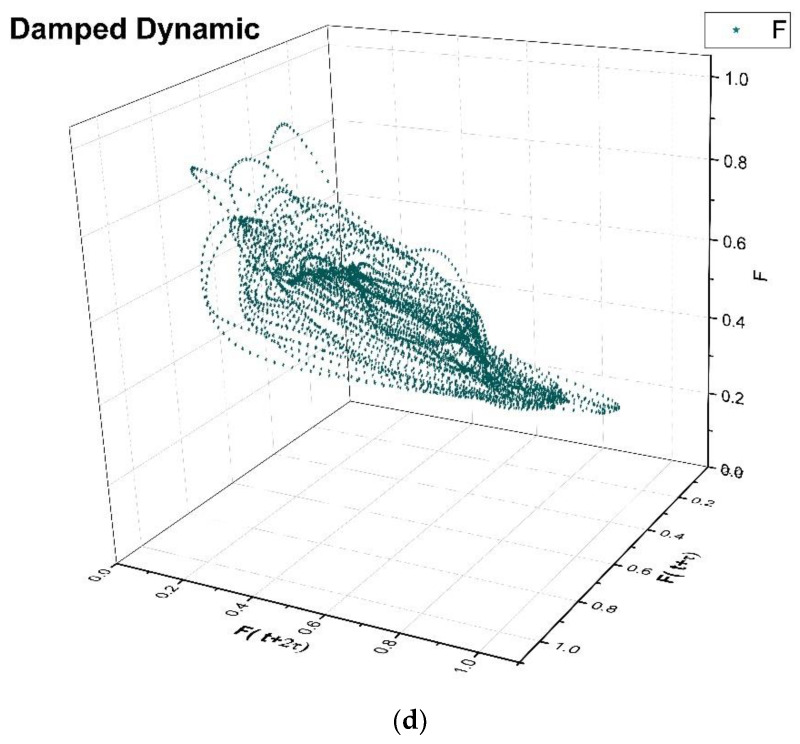
(**a**–**d**) Three-dimensional diagram (**a**), contour diagram (**b**), time series (**c**), and reconstituted attractor (**d**) in dimensionless coordinates of $\left|h\right|$ through harmonic mapping of damped complex systems dynamics.

**Figure 2:**
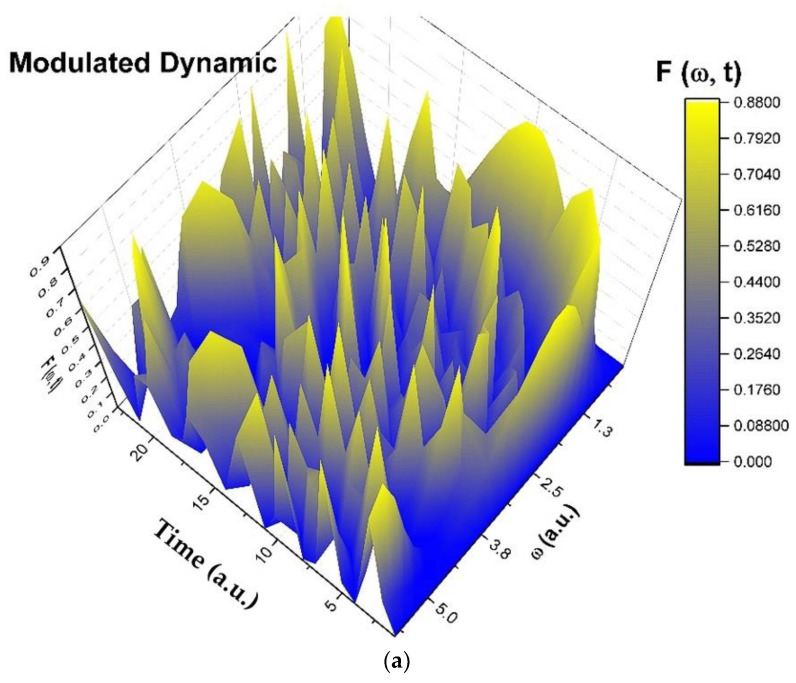

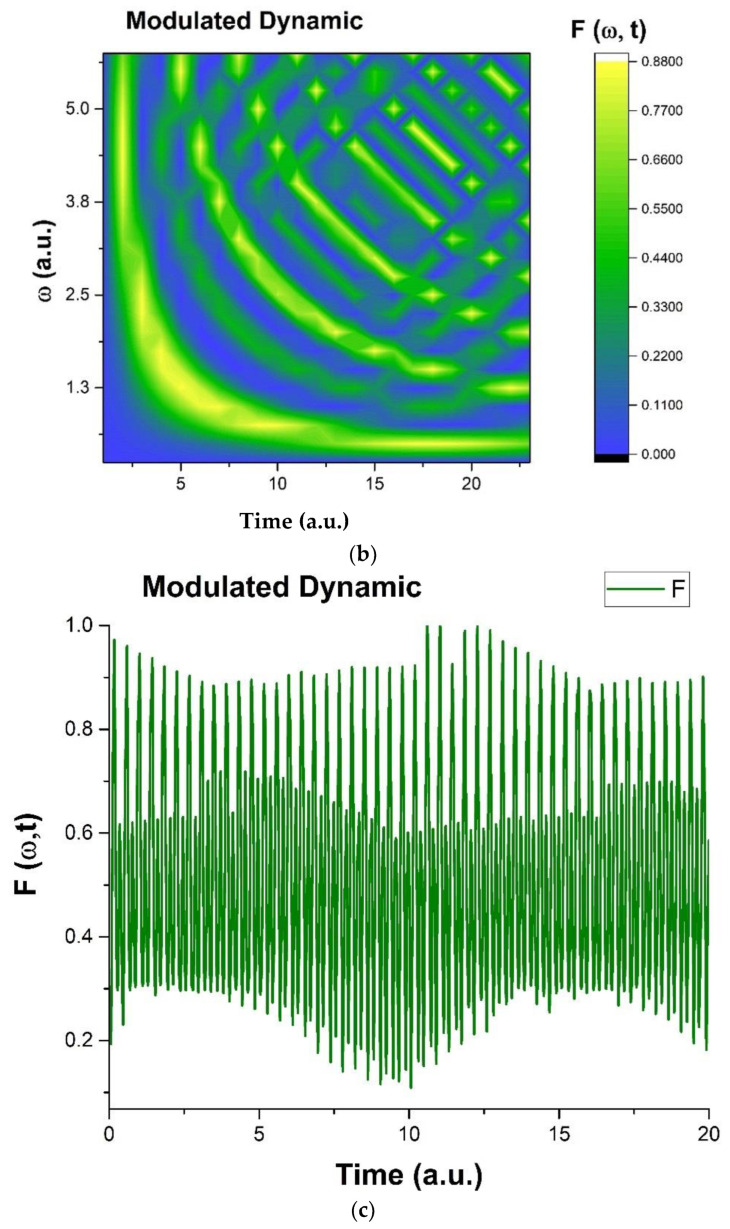

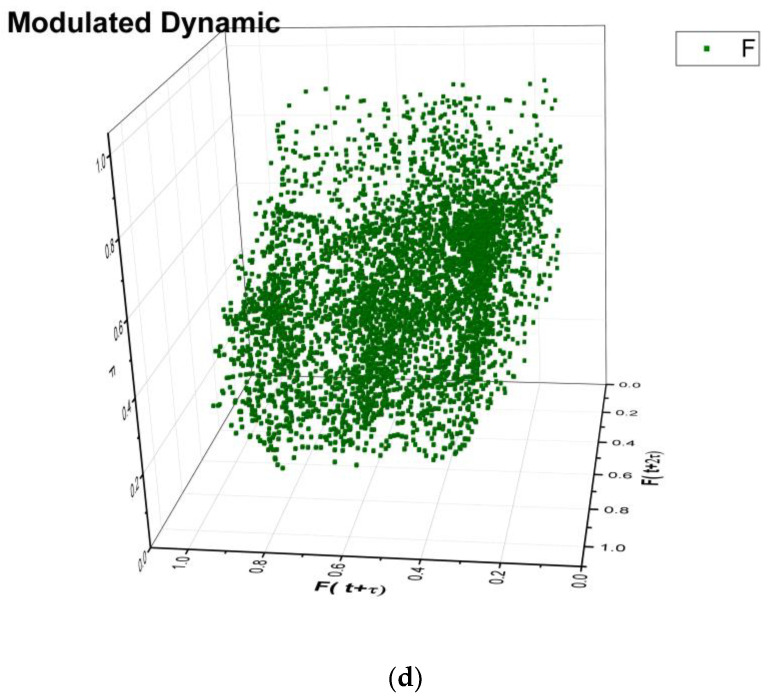
(**a**–**d**) Three-dimensional diagram (**a**), contour diagram (**b**), time series (**c**), and reconstituted attractor (**d**) in dimensionless coordinates of $\left|h\right|$ through harmonic mapping of modulated complex systems dynamics.

For α=ωt, transitions from stationary to non-stationary states in complex systems dynamics can be highlighted. We present in Figure 1a–d and Figure 2a–d harmonic mappings of damped and modulated complex systems dynamics. Because *h* can also be written as

$$h=\frac{i\left[e^{2\Phi }\sin\left(2\Omega t\right)-\sin\left(2\Omega t\right)-2ie^{\Phi }\right]}{e^{2\Phi }\left[\cos\left(2\Omega t\right)+1\right]-\cos\left(2\Omega t\right)+1}$$

we plotted $\left|h\right|$ for ϕ=2.35.

## 5. A Short Description of Gravity for an Axial Symmetry ERNST Potential

We are talking here about gravity as it is presented to us in the formalism of the Theory of General Relativity (Einstein’s field equations). Rarely, one can give a general solution of these equations. However, the vacuum and electromagnetic vacuum equations have such a solution that can be brought to a simpler form in the case of stationary metrics. Frederic J. Ernst was the one who pointed out this form [22,23] for the case of the axially symmetric field. Later, both he, but especially Israel and Wilson [24], whom we will follow here, have shown that it is possible to treat the general stationary case in a completely analogous way. We will follow this last work here, first because it seems a bit more explicit for what we want to bring out into evidence, then because it apparently has a fresh idea of bypassing the related indeterminacy of the metric tensor, leading to profitable results for mathematical philosophy. We still follow the general idea of Ernst’s original works, namely, that of posing the problem of the gravitational field in connection with a variational principle, for reasons that will be immediately highlighted.

The main point of the cited work of Israel and Wilson is that, for a stationary space–time metric, conveniently written in the form

(35) $$(ds{)}^{2}=f{\left(dx^{4}+\omega_{m}dx^{m}\right)}^{2}-f^{-1}\left(\gamma_{mn}dx^{m}dx^{n}\right)$$

where we use the convention of summation by repeated indices of different variance, Einstein’s equations for the electromagnetic vacuum field

(36) $$G_{\alpha \beta }=-8T_{\alpha \beta }$$

take the form of the system of equations with nonlinear partial derivatives

(37) $$\begin{array}{l} \nabla^{2}\epsilon =\nabla \epsilon \cdot \left(\nabla \epsilon +2\Psi^{*}\nabla \Psi \right)\\ f\nabla^{2}\Psi =\nabla \Psi \cdot \left(\nabla \epsilon +2\Psi^{*}\nabla \Psi \right) \end{array}$$

Let us note that in Equations (35) and (36), Greek indices go from 1 to 4, while Latin indices go from 1 to 3 and represent spatial indices.

In Equation (36), we used the convention from [22,23,24] (the Newton constant and the speed of light in a vacuum are identical with the unit).

The spatio-temporal metric tensor is defined by

$$g_{44}=f,\;\gamma_{mn}=g_{4m}g_{4n}-g_{44}g_{mn},\omega_{k}=\frac{g_{4k}}{g_{44}}$$

and for raising and lowering the spatial indices, the metric $\left(\gamma_{mn}\right)$ is used. All these components do not depend on the time coordinate (stationarity property). A potential four-vector $\left(A,A_{4}\right)\equiv \left(A_{\gamma }\right)$ describes the electromagnetic field, whose intensities are given by its covariant curl:

(38) $$F_{\alpha \beta }=\nabla_{\alpha }A_{\beta }-\nabla_{\beta }A_{\alpha }$$

This electromagnetic field satisfies the equation

(39) $$-4\pi T_{\mu \nu }\equiv g^{\alpha \beta }F_{\mu \alpha }F_{\nu \beta }-\frac{1}{4}g_{\mu \nu }F^{\alpha \beta }F_{\alpha \beta }$$

In Equation (39), we used the same convention as in the case of Equation (36).

Further, $G_{\alpha \beta }$ is the Einstein tensor associated with the metric field and defined by

(40) $$G_{\mu \nu }\equiv R_{\mu \nu }-\frac{1}{2}g_{\mu \nu }R$$

with $R_{\alpha \beta }$ the Ricci tensor of the metric and R the scalar invariant of the curvature. In relation to these symbols, then are defined the functions

(41) $$\Psi \equiv A_{4}+i\Phi ;\epsilon \equiv f-\Psi^{*}\Psi +i\phi ;i=\sqrt{-1}$$

where Φ ia a magnetic potential and ϕ an arbitrary function. Once we know the functions ϵ,Φ, and ϕ, we can construct the Ricci tensor corresponding to the metric $\left(\gamma_{mn}\right)$ by

(42) $$\begin{array}{c} -f^{2}R_{mn}(\gamma )=\frac{1}{2}\epsilon_{(m}\nabla_{n)}\epsilon^{*}+\Psi \nabla_{(m}\epsilon \cdot \nabla_{n)}\Psi^{*}\\ +\Psi^{*}\nabla_{(m}\epsilon^{*}\cdot \nabla_{n)}\Psi -\left(\epsilon +\epsilon^{*}\right)\nabla_{(m}\Psi \cdot \nabla_{n)}\Psi^{*} \end{array}$$

where the parentheses mean symmetrization in relation to the indices they contain.

F. J. Ernst [22] introduced the complex potential ϵ for the special case of the gravitational field with axial symmetry. It was later proven that spatial symmetry is not a necessary condition for the existence of such a potential [23], but only the stationarity of the metric field (time independence). In order to find a solution for the gravitational potentials (metric tensor components in Einstein’s sense [24]), it is necessary to solve Einstein’s Equation (36). In such a context, since the right side of these equations contains the energy tensor, an a priori knowledge of the metric tensor is required.

This problem has been repeatedly iterated in gravitational field physics, in one way or another, and among its settlement cases, there are some remarkable for their contribution to knowledge of the nature of the gravitational field [25,26,27,28]. Let us note that the problem of the gravitational field could be solved if we take a different approach than the usual one, in the sense of allowing the metric γ to be arbitrary, so that it can be conveniently chosen. Indeed, Israel and Wilson [23] note that Equation (42) should only be taken as compatibility conditions between a specially chosen spatial metric and the complex fields ϵ and Ψ. In the particular case of the ordinary Euclidean space, the conditions of compatibility are simply reduced to the linear equation

(43) $$\Psi =a+b\epsilon ,ab^{*}+a^{*}b=-\frac{1}{2}$$

and the whole construction comes back to solving the Laplace’s equation

(44) $$\nabla^{2}\xi =0,\xi \equiv (1+\epsilon {)}^{-1}$$

Through Equation (43), the gravitational field determines an electromagnetic field. Moreover, Ernst himself [22] noted the fact that a functional relationship between the complex gravitational and electromagnetic potentials solves the gravitational field problem.

In 1971, Ernst [23] proved that the theory providing Equations (37) and (42) above, applied to the purely gravitational case, can be obtained from the variational principle

(45) $$\delta ∭\;\left\{R(\gamma )+2\frac{\gamma^{mn}\left(\nabla_{m}\epsilon \right)\left(\nabla_{n}\epsilon^{*}\right)}{{\left(\epsilon +\epsilon^{*}\right)}^{2}}\right\}\sqrt{\det(\gamma )}\left(d^{3}x\right)=0$$

where R(γ) is the scalar curvature of the metric γ. As such, it can now be seen that in a Euclidean space, this variational principle refers exclusively to Ernst’s complex potential:

(46) $$\delta ∭\;\left\{\frac{\gamma^{mn}\left(\nabla_{m}\epsilon \right)\left(\nabla_{n}\epsilon^{*}\right)}{{\left(\epsilon +\epsilon^{*}\right)}^{2}}\right\}\left(d^{3}x\right)=0$$

In other words, only in cases where the gravitational field defines an electromagnetic field through a linear relationship of the type (43) can that gravitational field be described exclusively through a complex potential. Here, we will limit ourselves to this last case of the gravity field in a vacuum. The line of ideas that we have just presented opens a specific path for the solution of the problem of vacuum fields, because the variational principle (46) can be constructed in connection with the continuous group SL(2,R) that we have here in mind.

Richard Matzner and Charles Misner observed [28] that the principle variational (46) is actually a response to what constitutes the problem of harmonic applications (discussed by Misner in [29]). From this point of view, Equation (46) describes a harmonic application from Euclidean space to SL(2,R). This fact is evident if, instead of the Ernst ϵ potential, we use as a field variable h≡iϵ, so that Equation (46) becomes

(47) $$\delta ∭\;\left\{\frac{\gamma^{mn}\left(\nabla_{m}h\right)\left(\nabla_{n}h^{*}\right)}{{\left(h-h^{*}\right)}^{2}}\right\}\left(d^{3}x\right)=0$$

This variational equation describes a harmonic application between the usual Euclidean space of the metric $\left(\gamma_{mn}\right)$ and the higher complex plane (the Poincaré representation of the hyperbolic plane) with the metric given by

$$(ds{)}^{2}=-4\frac{(dh)\left(dh^{*}\right)}{{\left(h-h^{*}\right)}^{2}}\equiv \frac{(du{)}^{2}+(dv{)}^{2}}{v^{2}},h=u+iv$$

known as the invariant metric of the SL(2,R) group.

In this context, the complex potential h could gain physical meaning. Indeed, Equation (41) gives us for the case of the null electromagnetic field (pure gravitational field):

(48) h=−ϕ+if

so that the real part of the potential is arbitrary, while the imaginary part

(49) $$v\equiv f=g_{44}$$

is positive. These are essential qualities required by the geometry of the Poincaré half-plane. By this fact, the Poincaré metric is physically legitimized. Another attractive theoretical point of this potential is that the differential equations corresponding to the variational principle (47), known as the “Ernst equations” of the problem, take the shape

(50) $$\left(h-h^{*}\right)\left(\nabla^{2}h\right)=2(\nabla h)(\nabla h)\;\left(h-h^{*}\right)\left(\nabla^{2}h^{*}\right)=2(\nabla h^{*})(\nabla h^{*})$$

and complex conjugate, obviously, and can be easily solved with the help of Laplace’s equation. More precisely, the solution of Equation (32) can be written in the form

(51) $$h=-i\frac{cosh\psi -e^{-i\alpha }sinh\psi }{cosh\psi +e^{-i\alpha }sinh\psi },\nabla^{2}\psi =0$$

with α real and arbitrary. Therefore, here, the solution to the problem of the stationary gravitational field is also reduced to solving Laplace’s equation in the usual space of our experience, just like in classical Newtonian theory [30,31,32].

## 6. Conclusions

In this paper, correlations between the Space–Time Theory and the Scale Relativity Theory have been established. In this way, non-differentiable implementations in complex systems dynamics become operational. This could specify the fact that, even in the case of standard theories (General Relativity and Quantum Mechanics), several implications of a holographic-type principle, fundamental to modern theories, could be found. Precisely, the presence of the same type of SL(2R) symmetry can lead to possible correspondences between the Scale Relativity Theory and the General Relativity Theory. We want to note the fact that the Scale Relativity Theory can be reduced, for dynamics in the fractal dimension *D_F_* = 2 at Compton-scale resolution, to classical Quantum Mechanics, and, also, that the General Relativity Theory, for a gravitational field with axial symmetry, can be reduced to a complex Ernst-type potential. Because these two theories accept the same type of symmetry, non-differentiable implementations in the description of complex systems dynamics can be highlighted.

## Data Availability

Not applicable.
